# Uniform Movement for Dealing with Tuberculosis

**Published:** 1895-10

**Authors:** 


### Uniform Movement for Dealing with Tuberculosis.

A permanent organization of representatives of cattle commissions was organized at Boston, Massachusetts, in July last, with Dr. F. H. Osgood, President, Boston; Secretary and Treasurer, C. M. Winslow. Executive Committee: C. A. Dennen, Massachusetts; Clifton Polk, Connecticut; N. J. Batchelder, New Hampshire; John M. Deering, Maine; V. I. Spear, Vermont; and Obadiah Brown, Rhode Island.

Recommendations were adopted to secure uniform regulations to govern the movements of cattle from State to State, and the issuance and requirement of certificates of inspection. All animals shipped into the State to be held for six days and subjected to tuberculin-test, and if diseased to be destroyed without compensation. It was voted that the tuberculin-test was the only practicable test and that physical examination alone is useless, and that such tests be made only by competent veterinarians.

Connecticut, after September 1st, will allow half compensation for diseased animals. New Hampshire, having exhausted her appropriation, is unable to do anything further at present. Maine claims to be free from tuberculosis, and will not allow the entrance of any animals in the State unless they have been tested with tuberculin. Vermont exacts rigid measures, and in 3000 cases tested tuberculin has proven a reliable agent. New Jersey is anxious to move against the disease, specially because of her geographical situation.

Incompetent veterinarians will be placed on record and furnished the various State commissions for reference. It was recommended that the compensation to experts and agents be paid according to ability, with prospects of promotion.

---

PROSECUTION BY THE MARYLAND VETERINARY EXAMINING BOARD.—W. C. Sigmund, Jr., of 1224 East Lexington Street, Baltimore, was prosecuted under warrant issued on complaint of Dr. A. W. Clement, Secretary of the Board, for illegally practising veterinary medicine and surgery. The defendant was graduated from the National Veterinary College at Washington, this spring. This college requires but two sessions, while the law requires that all applicants for registration must be graduates of a school or college of veterinary medicine requiring three full sessions before issuing a diploma. As this is the first case to be tried under the law, the progress will be watched with interest. The defendant waived examination before the magistrate and asked for a jury trial. He was released on bail.

The Wyoming Valley Veterinary Medical Association contemplate changing their meetings from monthly to quarterly, to increase the attendance and interest, as many of the members live at quite distant points.

Another school for the teaching of horseshoeing is under consideration, to be established at Toronto, Canada.

